# Dipeptidyl peptidase-4 inhibitors and cancer risk in patients with type 2 diabetes: a meta-analysis of randomized clinical trials

**DOI:** 10.1038/s41598-017-07921-2

**Published:** 2017-08-15

**Authors:** Ming Zhao, Jiayi Chen, Yanyan Yuan, Zuquan Zou, Xiaolong Lai, Daud M Rahmani, Fuyan Wang, Yang Xi, Qin Huang, Shizhong Bu

**Affiliations:** 1 Runliang Diabetes Laboratory, Diabetes Research Center, School of Medicine, Ningbo University, 315211 Ningbo, China; 2 Department of Public Health, Longsai Hospital, 315200 Ningbo, China; 3 Department of Endocrinology, Changhai Hospital, Second Military Medical University, 200433 Shanghai, China

## Abstract

Some recent studies have suggested that the use of dipeptidyl peptidase-4 inhibitors (DPP4i) is associated with cancer development. However, some other studies suggest no such association. The aim of the present study was to evaluate the effect of DPP4i on the risk of developing cancers. The electronic databases PubMed, Medline, EMBASE, Web of Science and Cochrane Library and the clinical trial registry were searched for published and unpublished randomized clinical trials on humans. Eligible studies were RCTs conducted in patients with type 2 diabetes mellitus, comparing DPP4i with a placebo or other active drugs. A total of 72 trials with 35,768 and 33,319 patients enrolled for DPP4i and the comparison drugs, respectively. Overall, no significant associations were detected between the use of DPP4i and cancer development, in comparison with the use of other active drugs or placebo. The results were consistent across pre-defined subgroups stratified by type of DPP4i, type of cancer, drug for comparison, trial duration, or baseline characteristics. The results of this meta-analysis suggest that patients with type 2 diabetes treated with DPP4i do not have a higher risk of developing cancers than patients treated with a placebo or other drugs.

## Introduction

Diabetes is one of the serious public health problems of the 21^st^ century. The International Diabetes Federation estimated that the number of people with diabetes was 415 million, and it will reach 642 million by 2040^1^. In high-income countries, approximately 87% to 91% of all people with diabetes have type 2 diabetes^2–5^. Glucagon-like peptide-1 (GLP-1) and glucose-dependent insulinotropic polypeptide, also called gastric inhibitory polypeptide (GIP), are the two main physiological incretins synthesized in the intestinal tract and play an important role in the regulation of blood glucose^6^. GLP-1 inhibits the release of glucagon, reduces postprandial hepatic glucose generation and delays gastric emptying, which results in decreased postprandial glucose absorption^7^. Because these incretins are rapidly degraded by the enzyme dipeptidyl peptidase 4, their half-lives are short (GLP-1 1–2 minutes, GIP 7 minutes)^8^. Oral dipeptidyl peptidase-4 inhibitors (DPP4i), which reduce the release of GLP-1 and extend its half-life, have become relatively new incretin-based agents for treating type 2 diabetes^9^. Presently, there are over 10 DPP4i approved for clinical use, with several of them extensively studied, including data regarding malignancy outcomes, namely, sitagliptin, vildagliptin, saxagliptin, linagliptin and alogliptin, and they are currently recommended by international and national guidelines worldwide.

However, the long-term effect of DPP4i for the treatment of type 2 diabetes has been debated. Because the major complication in patients with type 2 diabetes is cardiovascular disease, the focus of many studies was to evaluate the cardiovascular safety of DPP4i or whether DPP4i could lower cardiovascular risk^10–12^. In addition, the association between DPP4i and cancer has been studied by many researchers. An analysis based on the US Food and Drug Administration (FDA) adverse event reporting system (AERS) database reported increased rates of pancreatic cancer with the use of sitagliptin compared with other anti-diabetes drugs. The reported event rate for pancreatic cancer was 2.7 times higher with sitiagliptin than other therapies (p = 0.008)^13^. Type 2 diabetes is an independent risk factor of colon cancer^14^, but whether DPP4i therapy affects the development of colon cancer has not been well investigated. Two large multicenter randomized controlled trials (RCTs), Saxagliptin Assessment of Vascular Outcomes Recorded in Patients with Diabetes Mellitus-Thrombolysis in Myocardial Infarction 53 (SAVOR-TIMI 53) and Trial Evaluating Cardiovascular Outcomes with Sitagliptin (TECOS), were conducted to assess the cardiovascular safety of saxagliptin and sitagliptin, respectively^11,12^. The results of the two trials indicated that there was no significant increase in the risk of pancreatic cancer. Interestingly, a protective effect of saxagliptin against colon cancer was found in the SAVOR-TIMI 53 trial (hazard ratio = 0.51, 95% CI = 0.27–0.92, p = 0.026)^15^.

There have been many RCTs to assess the efficacy and safety of DPP4i in diabetic patients. A meta-analysis conducted by Monami *et al*.^16^ had evaluated the effect of DPP4i on the developing pancreatic cancer in 2014. However, currently no published meta-analysis on the association between DPP4i and the risk of other types of cancers in patients with type 2 diabetes was reported. Given that previous published meta-analyses may not extract data from those completed and private RCTs, besides, some new clinical trials were published recently. Therefore, the purpose of this meta-analysis of RCTs was to systematically and comprehensively evaluate the effect of DPP4i use on the risk of developing cancers.

## Results

### Study Search

**Figure 1:**
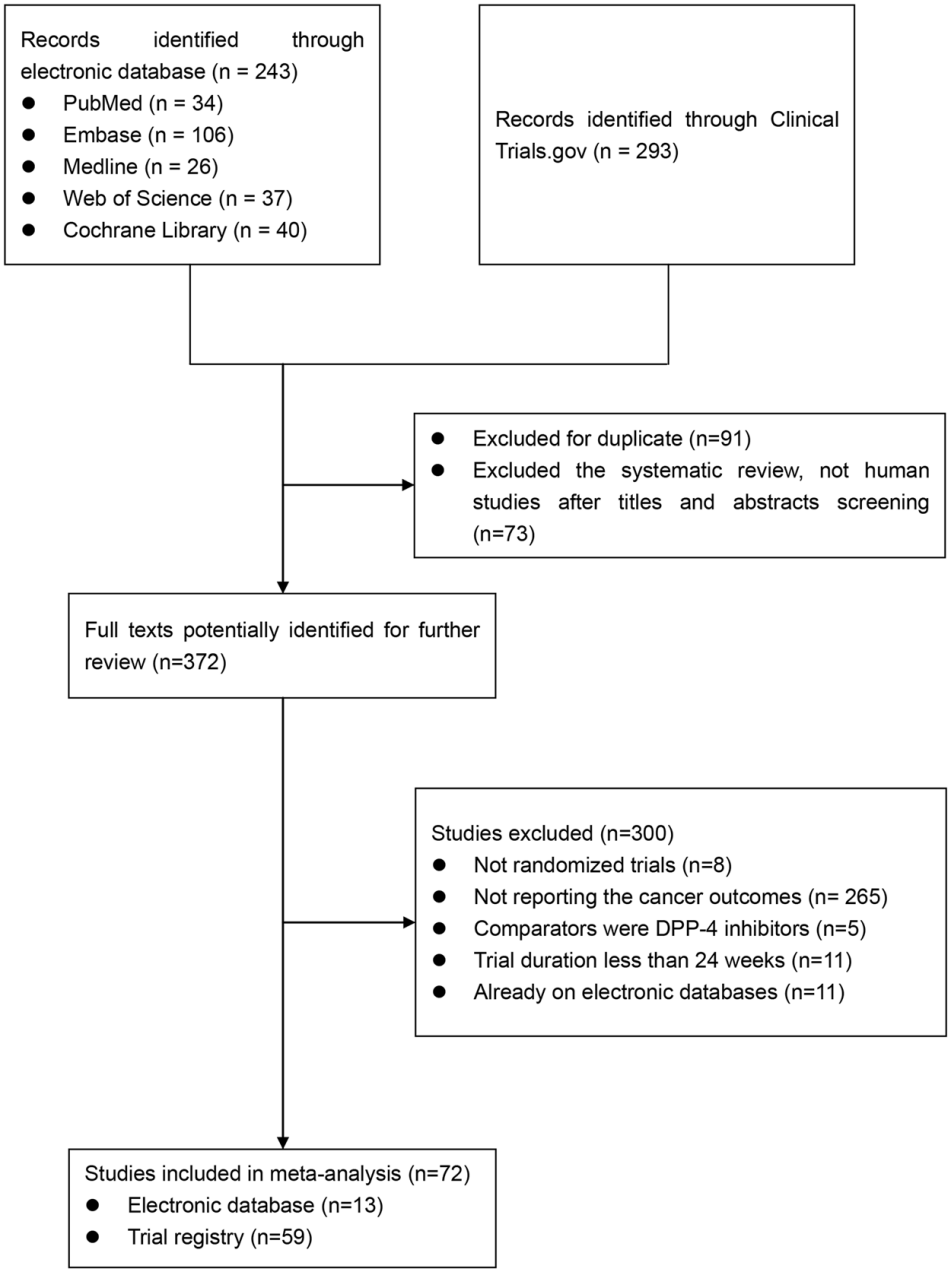
Flowchart of study selection process. DPP-4: dipeptidyl peptidase-4.

A total of 536 studies were identified through a search of the electronic databases and the www.clinicaltrials.gov website. After excluding duplicate publications, 445 studies remained. After the titles and abstracts were reviewed, 73 studies were excluded because they were systematic reviews or not human studies, leaving 372 studies for full text evaluation. Among them, 300 studies were excluded because of the following reasons: 8 studies were not randomized trials, 265 studies did not report cancer outcomes, in 5 studies the drugs for comparison were also DPP4i, 11 studies had a duration shorter than 24 weeks, and 11 studies reported on the website were published in the electronic databases, given the quality score of the studies, we excluded those studies both in the literature and in the registry from the trial registry. As a result, 72 RCTs, including 13 from electronic database and 59 from the trial registry were selected in the final meta-analysis. The details of the study search flow were shown in Fig. 1.

### Study Characteristics

Table 1 summarizes the study characteristics of the 72 trials totaling 69,087 patients, with 35,768 received various DPP4i (sitagliptin, saxagliptin, alogliptin and linagliptin) and 33,319 received other active anti-diabetic drugs or placebo^11,17–29^. The DPP4i tested were sitagliptin in 38 trials, saxagliptin in 13, alogliptin in 10, and linagliptin in 11. The adverse events of cancers were classified into 10 types based on the location of the cancer: 80 studies included reproductive system cancers, 89 included digestive system cancers, 30 included integumentary system cancers, 32 included urinary system cancers, 13 included hematologic system cancers, 23 included respiratory system cancers, 5 included motor system cancers, 17 included endocrine system cancers, 2 included nervous system cancers and 2 included cardiovascular system cancers. The mean age of the included patients ranged from 49.7 to 74.9 years. The sample sizes of individual trials were between 21 and 16492 patients and the duration of trial ranged from 24 weeks to 5 years. Among all of the patients included, the mean HbA1c was 8.0 ± 1.5%, mean BMI was 29.9 ± 2.3 kg/m^2^ and mean duration of diabetes was 6.9 ± 3.7 years.

**Table 1:** Characteristics of included randomized controlled trials.

Study	NCT code	Comparison drug(s)	No. of patients		Trial duration (weeks)	Males/Females	Mean Age (years)	HbA1c (%)	BMI (kg/m^2^)	DM duration (years)	Cancer type
			DPP-4i	Control							
**Sitagliptin**											
NCT01590771	NCT01590771	Placebo	249	249	24	249/249	57	8.55	NA	NA	Cervix carcinoma
NCT01189890	NCT01189890	Glimepiride	241	239	30	202/278	70.7	7.78	29.7	8.69	Colon cancer, Malignant melanoma, Prostate cancer
NCT01076088	NCT01076088	Placebo	120	127	24	161/86	52.7	8.86	25.7	1.1	Renal cancer
NCT00420511	NCT00420511	Placebo	10	11	48	14/7	60.9	6.1	32.7	3	Renal cancer
NCT01076075	NCT01076075	Placebo/Pioglitazone	210	212	24	193/229	54.9	8.4	28.5	7.75	Prostate cancer
Arjona 2013	NCT00509262	Glipizide	210	212	54	170/252	64.2	7.8	26.76	10.4	Breast cancer, Leukemia, Lung cancer, Pancreatic cancer
Green 2015	NCT00790205	Placebo	7332	7339	5 years	10374/4297	65.5	7.2	30.2	11.6	Gastric cancer, Colon cancer, Salivary gland neoplasm, Cervix carcinoma, Bladder cancer, Bone cancer, Breast cancer, Pancreatic cancer, Prostate cancer, Rectal cancer, Renal cancer, Bronchial carcinoma, Lung cancer, Thyroid cancer, Ovarian cancer, Endometrial cancer, Gallbladder cancer, Hepatic cancer, Bile duct cancer, Laryngeal cancer, Anal cancer, Leukemia, Lymphoma, Lip carcinoma
NCT00885352	NCT00885352	Placebo	157	156	26	195/118	56	8.8	29.9	9.8	Basal cell carcinoma
NCT00509236	NCT00509236	Glipizide	64	65	54	77/52	59.5	7.8	26.8	17.5	Prostate cancer
NCT00095056	NCT00095056	Placebo	65	26	54	47/44	67.9	7.7	26.6	13.5	Colon cancer, Pancreatic cancer, Prostate cancer, Skin cancer
NCT00395343	NCT00395343	Placebo	322	319	24	326/315	57.8	8.7	31	12.5	Breast cancer, Rectal cancer
NCT00722371	NCT00722371	Pioglitazone	231	230	54	249/212	52.03	8.8	31.3	4.1	Bladder cancer, Breast cancer, Malignant melanoma, Skin cancer
NCT00337610	NCT00337610	Placebo	96	94	30	88/102	54.8	9.2	30.2	NA	Pancreatic cancer, Colon cancer, Lymphoma
NCT01177384	NCT01177384	Placebo	191	189	24	194/186	57.1	8.08	NA	NA	Breast cancer, Thymoma
NCT00305604	NCT00305604	Placebo	102	104	24	97/109	71.9	7.8	30.95	7.1	Basal cell carcinoma, Colon cancer, Malignant melanoma, Skin cancer
NCT00701090	NCT00701090	Glimepiride	516	519	30	563/472	56.3	7.5	30	6.8	Basal cell carcinoma, Lung cancer, Pancreatic cancer
NCT00449930	NCT00449930	Metformin	528	522	24	484/566	56	7.3	30.8	2.4	Lung cancer, Malignant melanoma
NCT00637273	NCT00637273	Pioglitazone	166	165	26	165/166	52.6	8.5	32	5.5	Thyroid cancer
NCT01137812	NCT01137812	Canagliflozin	378	377	52	422/333	56.5	8.1	31.6	9.6	Cervix carcinoma, Lung cancer, Oesophageal cancer, Uterine cancer
NCT01098539	NCT01098539	Albiglutide	246	249	52	266/229	63.3	NA	NA	NA	Brain cancer, Breast cancer, Malignant melanoma, Prostate cancer, Renal cancer
Aschner 2012	NCT00751114	Insulin glargine	253	227	24	246/234	53.6	8.5	31.09	4.5	Prostate cancer
NCT00106704	NCT00106704	Placebo/Pioglitazone	222	219	54	234/207	56	8.34	31	8.8	Lung cancer, Skin cancer
NCT00881530	NCT00881530	Metformin	56	56	78	57/55	57.55	8.09	29.3	NA	Breast cancer, Cervix carcinoma
NCT00482729	NCT00482729	Metformin	625	621	44	709/537	49.7	9.87	33.3	3.3	Colon cancer, Endometrial cancer, Laryngeal cancer, Lung cancer, Malignant melanoma, Renal cancer, Ovarian cancer, Prostate cancer
NCT00086502	NCT00086502	Placebo	175	178	24	196/157	56.2	8	31.5	6.1	Lung cancer
NCT00532935	NCT00532935	Pioglitazone	261	256	32	277/240	52.3	8.9	29.8	3.3	Basal cell carcinoma, Colon cancer, Renal cancer
NCT01046110	NCT01046110	Insulin degludec	222	225	26	262/185	55.7	8.9	NA	NA	Bladder cancer
NCT02008682	NCT02008682	Liraglutide	184	183	26	219/148	51.5	8.12	27.24	5.27	Thyroid cancer,Thymoma
Ahren 2014	NCT00838903	Placebo/Metformin	302	101	104	189/214	54.8	8.1	32.6	6	Thyroid cancer, Breast cancer, Lung cancer, Malignant melanoma, Prostate cancer, Rectal cancer, Renal cancer
NCT00094770	NCT00094770	Glipizide	588	584	104	694/478	56.7	7.7	31.2	6.35	Basal cell carcinoma, Bladder cancer, Breast cancer, Colon cancer, Lymphoma, Gastric cancer, Hepatic cancer, Malignant melanoma, Bone cancer, Oesophageal cancer, Prostate cancer, Rectal cancer, Renal cancer, Skin cancer, Uterine cancer
Pratley 2012	NCT00700817	Liraglutide	219	221	52	236/204	55	8.4	32.9	6.4	Pancreatic cancer, Renal cancer, Bile duct cancer, Breast cancer, Colon cancer
NCT00289848	NCT00289848	Placebo	352	178	28	306/224	50.9	8.74	25	2	Ovarian cancer
NCT00813995	NCT00813995	Placebo	197	198	24	200/195	54.6	8.5	25.3	6.85	Colon cancer, Lip carcinoma
NCT00094757	NCT00094757	Placebo/Pioglitazone	205	110	54	179/136	54.85	8	32	4.6	Breast cancer, Bone cancer, Pancreatic cancer, Thyroid cancer, Uterine cancer
NCT01519674	NCT01519674	Metformin	195	194	24	205/184	55.84	8.4	29.4	NA	Pancreatic cancer, Thyroid cancer
NCT00086515	NCT00086515	Placebo/Glipizide	464	237	104	400/301	54.5	8	NA	6.2	Lymphoma, Basal cell carcinoma, Bladder cancer, Breast cancer, Renal cancer, Thyroid cancer, Hepatic cancer, Lung cancer, Ovarian cancer, Pancreatic cancer, Thyroid cancer, Skin cancer
NCT01907854	NCT01907854	Liraglutide	204	202	27	242/164	56.4	8.3	NA	NA	Bladder cancer
Weinstock 2015	NCT00734474	Dulaglutide	315	304	104	297/322	53.7	8.1	31.21	7.1	Breast cancer, Colon cancer, Gastric cancer, Laryngeal cancer, Prostate cancer,Thyroid cancer, Uterine cancer
**Saxagliptin**											
NCT00327015	NCT00327015	Metformin	335	328	24	332/331	51.96	9.5	30.2	1.7	Pancreatic cancer
NCT00121641	NCT00121641	Placebo	106	95	24	101/100	53.91	7.95	31.65	2.41	Colon cancer, Cervix carcinoma, Malignant melanoma, Renal cancer
NCT00316082	NCT00316082	Placebo	71	74	24	72/73	54.9	NA	NA	NA	Uterine cancer, Pancreatic cancer, Hepatic cancer
NCT00121667	NCT00121667	Placebo/Metformin	191	179	24	199/171	54.72	8.1	31.4	6.5	Breast cancer, Uterine cancer
NCT00295633	NCT00295633	Placebo	186	184	24	174/196	52.34	8.3	30.05	5.15	Colon cancer
NCT00575588	NCT00575588	Glipizide	428	430	104	444/414	57.55	7.7	31.4	5.4	Bladder cancer, Colon cancer, Salivary gland neoplasm, Leukemia, Thyroid cancer, Bladder cancer, Bronchial carcinoma, Lung cancer, Malignant melanoma, Renal cancer, Breast cancer
NCT00757588	NCT00757588	Placebo/Insulin	304	151	24	188/267	57.2	8.7	32.3	11.9	Breast cancer, Pituitary tumor, Uterine cancer, Pancreatic cancer, Prostate cancer
NCT01128153	NCT01128153	Placebo	129	128	24	154/103	57	8.28	29.2	NA	Laryngeal cancer
Schernthaner 2015	NCT01006603	Glimepiride	360	360	52	445/275	72.6	7.6	29.6	7.6	Breast cancer, Basal cell carcinoma, Bladder cancer, Colon cancer, Hepatic cancer, Lung cancer, Pancreatic cancer, Prostate cancer, Salivary gland neoplasm
NCT00313313	NCT00313313	Placebo/Glyburide	253	267	24	233/287	54.96	8.45	29	6.8	Thyroid cancer
NCT01006590	NCT01006590	Metformin	147	139	24	164/122	58.7	7.8	31.7	6.5	Prostate cancer, Tongue cancer
Leiter 2016	NCT01107886	Placebo	8280	8212	2.9 years	11037/5455	65	8	31.1	10.3	Thyroid cancer, Bladder cancer, Breast cancer, Bronchial carcinoma, Cervix carcinoma, Colon cancer,Gastric cancer, Hepatic cancer, Lung cancer, Lymphoma, Ovarian cancer, Prostate cancer, Pancreatic cancer, Oesophageal cancer, Endometrial cancer, Leukemia, Lymphoma, Laryngeal cancer, Skin cancer, Vascular neoplasm, Bile duct cancer, Gallbladder cancer, Hodgkin’s disease, Malignant melanoma, Testis cancer, Bone cancer, Lip carcinoma, Haemangioma, Brain cancer, Rectal cancer, Renal cancer, Uterine cancer
NCT00374907	NCT00374907	Placebo/Metformin	20	16	116	14/22	55.5	NA	33.01	NA	Colon cancer
Alogliptin											
White 2013	NCT00968708	Placebo	2701	2679	41 months	3651/1738	61	7.2	28.7	7.2	Colon cancer, Prostate cancer, Breast cancer, Bladder cancer, Gastric cancer, Renal cancer, Lung cancer
NCT00286468	NCT00286468	Placebo	198	99	26	150/147	56.7	8.11	30	7.6	Basal cell carcinoma, Bladder cancer
NCT00286442	NCT00286442	Placebo	213	104	26	151/166	55.33	7.93	32	6	Endometrial cancer, Prostate cancer
NCT00856284	NCT00856284	Glipizide	878	869	104	881/866	55.4	7.61	31.2	5.45	Breast cancer, Basal cell carcinoma, Colon cancer, Bladder cancer, Endometrial cancer,Lymphoma, Ovarian cancer, Renal cancer, Lung cancer
NCT00286429	NCT00286429	Placebo	129	130	26	106/153	55.4	9.3	32.4	12.8	Cervix carcinoma
NCT01263496	NCT01263496	Voglibose	97	83	40	131/49	NA	NA	NA	NA	Pancreatic cancer, Breast cancer
NCT00395512	NCT00395512	Pioglitazone	164	163	26	166/161	52.1	8.78	31.47	3.14	Colon cancer
NCT00707993	NCT00707993	Glipizide	222	219	52	198/243	69.9	NA	29.79	6.1	Breast cancer, Cervix carcinoma
NCT00432276	NCT00432276	Metformin	404	399	52	414/389	55.1	8.15	31.55	7.16	Colon cancer
Mita 2016	NA	OAD	172	169	2 years	199/142	64.6	7.25	24.75	8.6	Thyroid cancer, Prostate cancer, Cholangioma
**Linagliptin**											
NCT01084005	NCT01084005	Placebo	162	79	24	165/76	74.9	7.78	29.67	NA	Lung cancer, Malignant melanoma, Prostate cancer
NCT01087502	NCT01087502	Placebo/Glimepiride	113	122	52	149/86	66.6	8.05	32.09	NA	Bladder cancer, Lung cancer, Basal cell carcinoma, Colon cancer, Prostate cancer
NCT01734785	NCT01734785	Placebo	606	110	24	NA	NA	NA	NA	NA	Colon cancer, Basal cell carcinoma, Bladder cancer
Bajaj 2014	NCT00996658	Placebo	183	89	24	132/140	53.79	8.42	28.27	NA	Colon cancer
NCT00954447	NCT00954447	Placebo	631	630	52	658/603	60	8.3	31	8.3	Bile duct cancer, Colon cancer, Gastric cancer, Hepatic cancer, Lung cancer, Ovarian cancer, Renal cancer, Uterine cancer
NCT00798161	NCT00798161	Placebo	142	72	24	116/98	56.03	8.69	28.84	NA	Breast cancer
Gallwitz 2012	NCT00622284	Glimepiride	776	775	2 years	933/618	59.8	7.7	30.25	5	Breast cancer, Bronchial carcinoma, Colon cancer, Endometrial cancer, Laryngeal cancer, Hepatic cancer, Bone cancer, Ovarian cancer, Pancreatic cancer, Prostate cancer, Rectal cancer, Renal cancer, Lung cancer, Thyroid cancer, Vulval cancer
NCT01215097	NCT01215097	Placebo	205	100	24	152/153	55.5	7.99	25.6	NA	Gastric cancer
NCT00621140	NCT00621140	Placebo	336	167	24	243/260	55.7	8	29.05	NA	Breast cancer, Lymphoma
NCT00654381	NCT00654381	Placebo	159	80	52	395/166	60	NA	NA	NA	Gastric cancer, Prostate cancer
Barnett 2012	NCT00740051	Placebo/Glimepiride	151	76	52	88/139	56.5	8.1	29.5	NA	Colon cancer

NA, not available; NCT: national clinical trial; DPP4i: dipeptidyl peptidase-4 inhibitors; OAD: oral antidiabetes drug (excluding other DPP-4 inhibitors, GLP-1 analogs, and insulin).

The quality assessment of the 72 RCTs is summarized in Table S1. Among these studies, 49 (68.06%) were conducted with appropriate randomization, allocation concealment, blinding and reporting, which met the Cochrane criteria risk of bias for low risk of bias. The remaining 23 studies (31.94%) studies had unclear risk of bias. All of the trials were funded by industrial companies except one, which was funded by Juntendo University Graduate School of Medicine^18^.

### Effect of DPP4i on the risk of overall cancers

**Figure 2:**
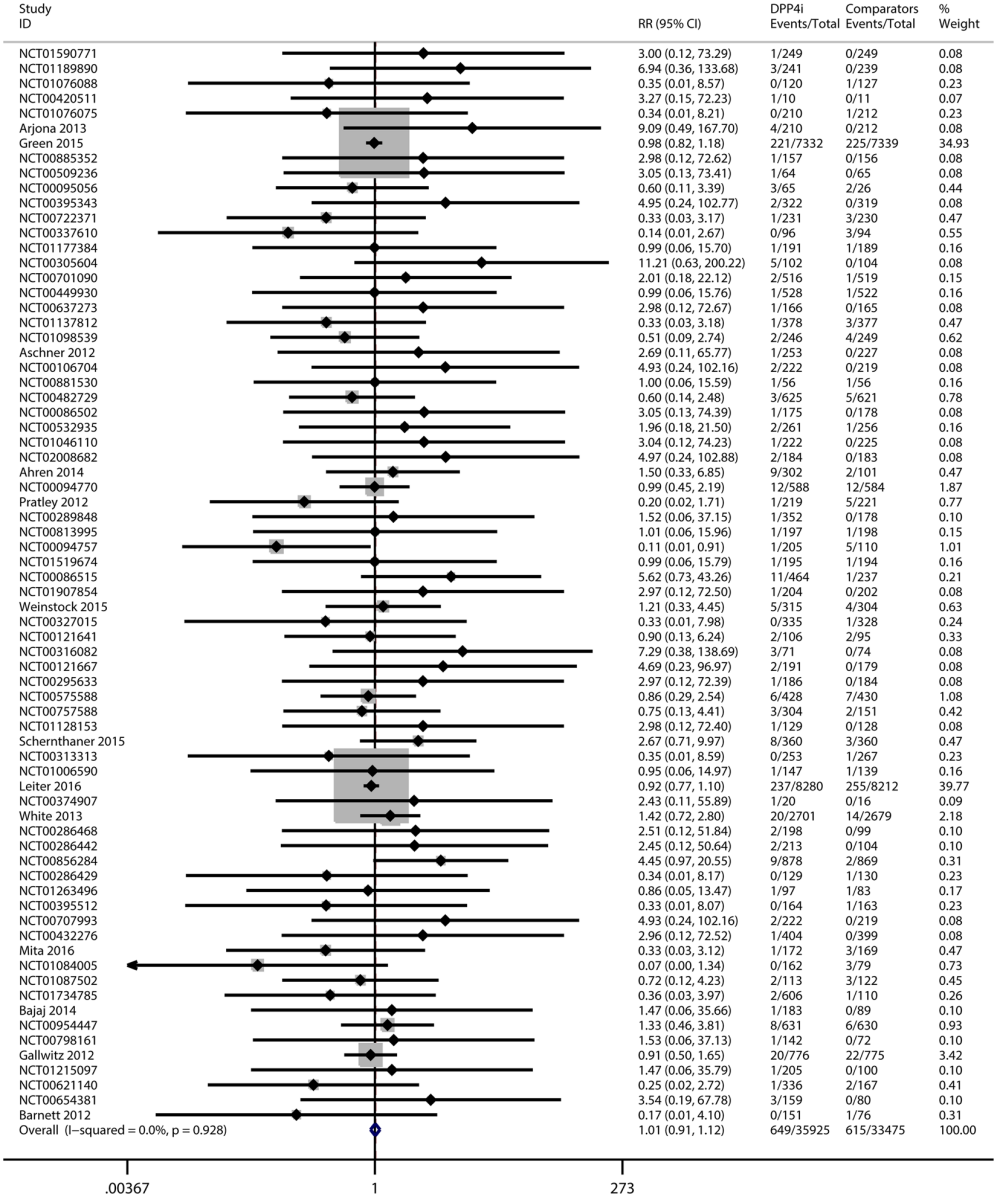
Risk of cancers in patients with type 2 diabetes who were treated with DPP-4 inhibitors versus other drugs. DPP4i: dipeptidyl peptidase-4 inhibitors.

The pooled RRs and 95% CIs were calculated to assess the effect of DPP-4i on the overall risk of developing cancers using a fixed-effect model. No significant associations were detected between DPP4i and cancer, compared with active drugs or placebo (MH-RR = 1.01, 95% CI = 0.91–1.12, *p* = 0.885). No heterogeneity was observed among these studies (*I* ^2^ = 0.0%, *p* = 0.928) (Table 2, Fig. 2).

**Table 2:** Meta-analysis results.

Variables	No. of studies	Pooled Relative Risk		Model of meta-analysis	Heterogeneity test	
		RR (95% CI)	*P*_*Z*_		*I* ^2^ (%)	*P*_*H*_
**Overall**	72	1.01 (0.91–1.12)	0.89	Fixed	0.0	0.93
**Type of DPP-4 inhibitors**						
Sitagliptin	38	1.03 (0.88–1.21)	0.68	Fixed	0.0	0.79
Saxagliptin	13	0.96 (0.81–1.13)	0.63	Fixed	0.0	0.81
Alogliptin	10	1.53 (0.93–2.53)	0.10	Fixed	0.0	0.68
Linagliptin	11	0.84 (0.55–1.28)	0.41	Fixed	0.0	0.70
**Type of cancers**						
Reproductive system	80	0.87 (0.73–1.05)	0.15	Fixed	0.0	1.00
Cervix carcinoma	8	1.38 (0.54–3.50)	0.50	Fixed	0.0	0.75
Prostate cancer	22	0.89 (0.69–1.14)	0.37	Fixed	0.0	0.97
Breast cancer	25	0.72 (0.50–1.06)	0.09	Fixed	0.0	0.98
Ovarian cancer	8	1.06 (0.48–2.35)	0.89	Fixed	0.0	0.87
Endometrial cancer	6	0.53 (0.22–1.28)	0.16	Fixed	0.0	0.70
Uterine cancer	9	1.77 (0.77–4.04)	0.18	Fixed	0.0	0.85
Vulval cancer	1	0.33 (0.01–8.16)	0.50	Fixed	—	—
Testis cancer	1	0.33 (0.01–8.11)	0.50	Fixed	—	—
Digestive system	89	0.93 (0.77–1.13)	0.46	Fixed	0.0	1.00
Colon cancer	27	0.96 (0.71–1.31)	0.81	Fixed	0.0	0.98
Pancreatic cancer	16	0.83 (0.51–1.35)	0.46	Fixed	0.0	0.93
Gastric cancer	8	1.35 (0.76–2.40)	0.30	Fixed	0.0	0.92
Salivary gland neoplasm	3	1.40 (0.28–7.10)	0.68	Fixed	0.0	0.55
Rectal cancer	6	0.41 (0.18–0.95)	0.04	Fixed	0.0	0.53
Gallbladder cancer	2	1.00 (0.14–7.06)	1.00	Fixed	0.0	0.34
Hepatic cancer	8	1.02 (0.54–1.91)	0.96	Fixed	0.0	0.85
Bile duct cancer	4	0.77 (0.28–2.12)	0.61	Fixed	0.0	0.60
Laryngeal cancer	6	0.71 (0.28–1.81)	0.47	Fixed	0.0	0.58
Anal cancer	1	0.20 (0.01–4.17)	0.30	Fixed	—	—
Lip carcinoma	3	4.33 (0.74–25.54)	0.11	Fixed	0.0	0.90
Oesophageal cancer	3	0.75 (0.17–3.33)	0.70	Fixed	0.0	0.58
Tongue cancer	1	0.32 (0.01–7.68)	0.48	Fixed	—	—
Cholangioma	1	2.95 (0.12–71.86)	0.51	Fixed	—	—
Integumentary system	30	1.12 (0.75–1.67)	0.58	Fixed	0.0	1.00
Malignant melanoma	12	0.87 (0.48–1.59)	0.66	Fixed	0.0	0.90
Basal cell carcinoma	11	0.95 (0.42–2.12)	0.90	Fixed	0.0	0.60
Skin cancer	7	1.79 (0.86–3.71)	0.12	Fixed	0.0	1.00
Urinary system	32	1.22 (0.92–1.61)	0.16	Fixed	0.0	0.98
Renal cancer	17	1.38 (0.89–2.13)	0.15	Fixed	0.0	0.96
Bladder cancer	15	1.12 (0.77–1.61)	0.56	Fixed	0.0	0.80
Hematologic system	13	1.05 (0.65–1.68)	0.85	Fixed	0.0	0.92
Leukemia	4	0.93 (0.45–1.93)	0.85	Fixed	0.0	0.74
Lymphoma	8	1.29 (0.67–2.49)	0.45	Fixed	0.0	0.88
Hodgkin’s disease	1	0.20 (0.01–4.13)	0.30	Fixed	—	—
Respiratory system	23	1.08 (0.82–1.41)	0.61	Fixed	0.0	0.91
Lung cancer	19	1.00 (0.76–1.33)	0.98	Fixed	0.0	0.95
Bronchial carcinoma	4	2.55 (0.86–7.59)	0.09	Fixed	0.0	0.55
Motor system	5	1.68 (0.57–5.01)	0.35	Fixed	0.0	0.64
Bone cancer	5	1.68 (0.57–5.01)	0.35	Fixed	0.0	0.64
Endocrine system	17	0.78 (0.43–1.41)	0.41	Fixed	0.0	0.91
Thyroid cancer	14	0.73 (0.38–1.40)	0.35	Fixed	0.0	0.91
Thymoma	2	2.98 (0.31–28.48)	0.34	Fixed	0.0	0.91
Pituitary tumor	1	0.17 (0.01–4.05)	0.27	Fixed	—	—
Nervous system	2	3.01 (0.31–28.84)	0.34	Fixed	0.0	0.99
Brain cancer	2	3.01 (0.31–28.84)	0.34	Fixed	0.0	0.99
Cardiovascular system	2	0.99 (0.14–7.04)	0.99	Fixed	0.0	0.34
Vascular neoplasm	1	2.98 (0.12–73.03)	0.50	Fixed	—	—
Haemangioma	1	0.33 (0.01–8.11)	0.50	Fixed	—	—
Type of comparators						
Placebo	30	0.98 (0.87–1.11)	0.79	Fixed	0.0	0.89
Sulfonylureas	14	1.40 (0.99–1.96)	0.05	Fixed	13.6	0.31
Thiazolidinediones	7	0.60 (0.27–1.33)	0.21	Fixed	11.6	0.34
Metformin	10	1.14 (0.57–2.28)	0.72	Fixed	0.0	1.00
SGLT inhibitors	1	0.33 (0.04–3.18)	0.34	Fixed	—	—
GLP-1 RAs	5	0.85 (0.39–1.83)	0.68	Fixed	6.3	0.37
Other antidiabetic agents	5	0.88 (0.32–2.42)	0.80	Fixed	0.0	0.77
Trial duration						
Less than 52 weeks	44	1.32 (0.93–1.89)	0.12	Fixed	0.0	0.98
52 weeks or more	28	0.98 (0.88–1.10)	0.73	Fixed	0.0	0.49
Mean age						
Less than 60 years	54	1.10 (0.85–1.40)	0.49	Fixed	0.0	0.97
60 years or more	16	0.99 (0.88–1.12)	0.89	Fixed	17.8	0.25
Mean HbA1c						
Less than 8%	21	1.06 (0.91–1.24)	0.43	Fixed	0.0	0.49
8% or more	44	0.94 (0.81–1.10)	0.45	Fixed	0.0	0.95
**Mean BMI**						
Less than 30 kg/m2	25	1.28 (0.88–1.86)	0.20	Fixed	0.0	0.80
30 kg/m2 or more	37	0.97 (0.86–1.10)	0.58	Fixed	0.0	0.87
**Mean diabetes duration**						
Less than 8 years	37	1.23 (0.96–1.58)	0.10	Fixed	0.0	0.80
8 years or more	14	0.97 (0.86–1.10)	0.61	Fixed	0.0	0.66

SGLT: sodium-glucose co-transporter; GLP-1 RAs: glucagon-like peptidase-1 receptor agonists.

### Subgroup analysis

**Figure 3:**
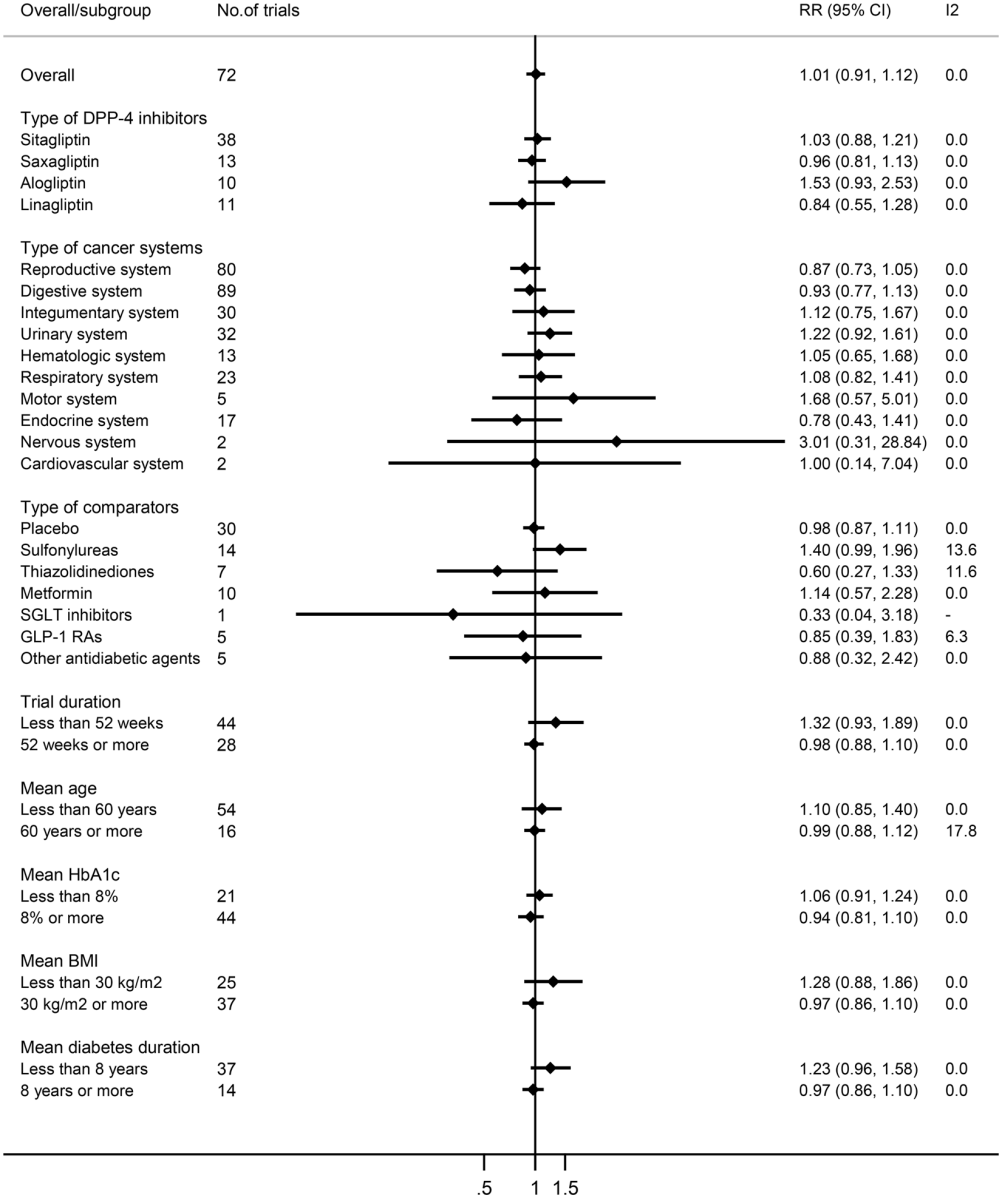
Overall and subgroup meta-analysis of DPP-4 inhibitors on risk of cancers.

Subgroup analysis was performed to determine whether the type of DPP4i, cancer subtype, drug for comparison, trial duration, or the baseline characteristics (age, HbA1c, BMI, or diabetes duration) had an effect on the RR of cancers with DPP4i (Table 2, Fig. 3).

### Effect of individual DPP4i on the risk of cancers

The risk of cancers (MH-RR) with individual DPP4i was 1.03 (95% CI = 0.88–1.21, *p* = 0.679) for sitagliptin (N = 38 trials), 0.96 (95% CI = 0.81–1.13, *p* = 0.627) for saxagliptin (N = 13 trials), 1.53 (95% CI = 0.93–2.53, *p* = 0.095) for alogliptin (N = 10 trials) and 0.84 (95% CI = 0.55–1.28, *p* = 0.410) for linagliptin (N = 11 trials). There was no statistically significant association between the risk of cancers and any of the individual DPP4i, with insignificant heterogeneity detected across these studies.

### Effect of DPP4i on the risk of individual cancers

We next preformed a subgroup analysis according to the different types of cancers.

A total of eighty studies examined the association between DPP4i and the risk of reproductive system cancers. The MH-RR of the reproductive system cancer risk with DPP4i was 0.87 (95% CI = 0.73–1.05, *p* = 0.152). Among all of the reproductive system cancers, prostate cancer and breast cancer were reported more frequently, 0.89 (95% CI = 0.69–1.14, *p* = 0.365) and 0.72 (95% CI = 0.50–1.06, *p* = 0.094), respectively. Eighty-nine studies assessed the association between DPP4i and the risk of digestive system cancers. The MH-RR of the digestive system cancer risk with DPP4i was 0.93 (95% CI = 0.77–1.13, *p* = 0.464). The result showed no significant association between DPP4i and colon cancer or pancreatic cancer risk (MH-RR = 0.96, 95% CI = 0.71–1.31; MH-RR = 0.83, 95% CI = 0.51–1.35, respectively). Analysis of twelve studies indicated that DPP4i were not associated with a significantly increased risk of developing malignant melanoma (MH-RR = 0.87, 95% CI = 0.48–1.59, *p* = 0.655). In addition, there were seventeen, fifteen, nineteen and fourteen studies on renal, bladder, lung and thyroid cancers, respectively. The results of MH-RR all showed that DPP4i were not associated with these cancers.

### Effect of type of comparison drug, trial duration and baseline characteristics on the risk of cancers

We performed analyses based on different types of comparison drugs, including placebo, sulfonylureas, thiazolidinediones, metformin, SGLT inhibitors, GLP-1 RAs and other antidiabetic agents. Among the studies, 30 used a placebo, 14 used sulfonylureas and 10 used metformin. There were no differences in the risk of cancers between DPP4i and any of these three comparison drugs (MH-RR = 0.98, 95% CI = 0.87–1.11, *p* = 0.789; MH-RR = 1.40, 95% CI = 0.99–1.96, *p* = 0.053; MH-RR = 1.14, 95% CI = 0.57–2.28, *p* = 0.718, respectively). Then we conducted a subgroup analysis stratified according to the duration of treatment. For a duration of less than 52 weeks, with 44 studies, no statistically significant difference was observed between patients in the DPP4i and comparison drug groups (MH-RR = 1.32, 95% CI = 0.93–1.89, *p* = 0.124). No significantly increased risk of digestive system cancers was observed for a duration of 52 weeks or more (MH-RR = 0.98, 95% CI = 0.88–1.10, *p* = 0.731). Moreover, subgroup analyses based on baseline characteristics were performed. Similarly, we found no significant differences in adverse events of cancers among studies with different mean age, mean HbA1c, mean BMI or mean diabetes duration.

### Sensitivity analysis and publication bias

Sensitivity analysis was performed to evaluate the stability of results among studies. The results of sensitivity analysis using alternative statistical method (random-effect model, MH-RR = 0.99, 95% CI = 0.89–1.11), effect measure (MH-OR = 1.01, 95% CI = 0.90–1.13), and excluding studies with more than three items with unclear risk of bias (MH-RR = 1.02, 95% CI = 0.88–1.18) did not show any significant changes in pooled effects.

**Figure 4:**
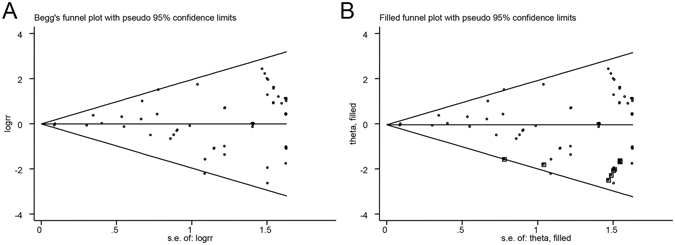
Funnel plot for the analysis of the effect of DPP-4 inhibitors on the risk of cancers. (**A**) Begg’s funnel plot of publication bias for the analysis of the pooled RRs. (**B**) The adjusted funnel plot using Trim and Fill method for publication bias.

Finally, publication bias was assessed by funnel plots, and the asymmetry of the funnel plots were evaluated by Begg’s and Egger’s tests. Based on the Begg’s test (*p* = 0.120), Egger’s test (*p* = 0.083), and that the funnel plot was asymmetrical on visual inspection, there was a possibility of publication bias. Because of this, we conducted a sensitivity analysis using the trim and fill method, based on the assumption that the funnel plot asymmetry was caused by publication bias, and used the iterative method to estimate missing studies those were negative unpublished. After removing the most extreme small studies from the positive side of the funnel plot and adding hypothetical studies, we re-calculated the pooled RR and 95% CI until the funnel plot was symmetric; if the changes were not statistically significant, publication bias had little effect on the results and the results were stable. The results of trim and fill method showed that there was no statistical significance between the risk of cancers and DPP4i (MH-RR = 0.95, 95% CI = 0.86–1.06) (Fig. 4).

## Discussion

DPP4i is a class of new drugs that treat type 2 diabetes effectively. The advantages of using DPP4i for diabetes therapy are their insignificance on body weight gain and the low risk of hypoglycemia. However, some potential adverse events have been detected in the clinic^30–33^. Currently, many studies focus on the effect of DPP4i on cardiovascular disease, bone fracture and heart failure^34–37^. However, cancer adverse events of DPP4i during the treatment of type 2 diabetes should not be neglected. The aim of the present study was to assess the association of DPP4i use and cancer risk in the patients with type 2 diabetes.

The current meta-analysis, which analyzed the results of 72 RCTs, indicated that there was no significantly increased risk of cancer associated with DPP4i (MH-RR = 1.01, 95% CI = 0.91–1.12, *p* = 0.885). Same results were found using pre-defined subgroup analyses that grouped the studies by type of DPP4i, cancer subtype, comparison drug, trial duration, and baseline characteristics (age, HbA1c, BMI, and diabetes duration). Our result was also in agreement with a previous meta-analysis conducted by Monami *et al*.^38^, which reported that no significant difference in cancer incidence was observed between DPP4i and the comparison drug groups (MH-OR = 1.020, 95% CI = 0.74–1.40, *p* = 0.90).

Many types of cancer were observed during the RCTs. We divided these cancers into several groups based on the location of the cancer. The number of some types of cancer was small, for instance, vulval cancer, testis cancer, anal cancer, tongue cancer and so on, causing the RR of cancer to be small and the confidence intervals of risk estimates to be large. With the currently available information, very few studies had specifically explored the association between cancers and DPP4i, except for pancreatic cancer, thyroid cancer and colon cancer. Although RCTs were considered as the most rigorous studies to clarify the cause-effect relationships between drug exposure and outcome via double-blind and randomized controlled method, due to financial limitations, and we will rely upon more pharamcovigilance studies to assess the risk of cancers with DPP4i.

We found no evidence of increased risk of pancreatic cancer in patients treated with DPP4i (MH-RR = 0.83, 95% CI = 0.51–1.35), which was inconsistent with an aforementioned study published by Elashoff *et al*.^13^, who examined the reports of AEs from FAD-AERS databases from 2004–2009, and calculated the OR between the reported cases using sitagliptin and other anti-diabetes drugs; they concluded a high risk of pancreatic cancer with sitagliptin. It is known that incomplete data and reporting bias existed in the FAD-AERS database^39^. Furthermore, the criteria of inclusion and exclusion were not standardized; for instance, patients with back pain, urinary tract infection, chest pain, cough, and syncope were included in the control group, those diseases may had an influence on the tumorigenesis^13^. The reason for the increased risk of pancreatic cancer with incretin-based, in the opinion of Elashoff *et al*., was that the GLP-1-based therapy could induce asymptomatic low-grade pancreatitis^40^, and pancreatitis was relevant to the cellular transformation that leads to pancreatic cancer^41^, whether the GLP-1-based therapy could lead to pancreatic cancer eventually was still under debate^42^. In the current study, we compared the effect of DPP4i versus different types of comparison drugs and the results all showed no significant increase in pancreatic cancer, among these, sulfonylureas (MH-RR = 1.40, 95% CI = 0.99–1.96, *p* = 0.053), thiazolidinediones (MH-RR = 0.60, 95% CI = 0.27–1.33, *p* = 0.209). A new-user cohort study conducted by Gokhale *et al*.^43^ reported that the hazard of pancreatic cancer with DPP4i was similar to that with thiazolidinediones (HR = 1.0, 95% CI = 0.7–1.4) but lower than that with sulfonyureas (hazard ratio (HR) = 0.6, 95% CI = 0.4–0.9). The limitation of this study was the short treatment duration, and also it was an observational study, so the confounding bias induced by other potential variables may affect the outcomes.

Insulin resistance had been suggested as one of the causes of thyroid cancer^44^. Therefore, the use of antidiabetic drugs may negatively affect the development of thyroid cancer. In our study, we found that there was no statistical significance in the association between DPP4i and thyroid cancer (MH-RR = 0.73, 95% CI = 0.38–1.40, *p* = 0.345). This was consistent with the result concluded from the FAD-AERS database, which showed that sitagliptin was associated with a higher but not significant risk of thyroid cancer compared with control drugs (OR = 1.48, *p* = 0.65)^13^. However, a recent study evaluated such association in Taiwanese patients with newly diagnosed type 2 diabetes from 1999 to 2008 by using the reimbursement database of the National Health Insurance, and found that the overall HR for patients newly treated with sitagliptin compared with those treated with other antidiabetic drugs was 1.516 (95% CI = 1.011–2.271), indicating that sitagliptin use was associated with an increased risk of thyroid cancer^45^. The reason of this discrepancy may be that the database from which the Taiwanese study extracted records from lacked data for potential confounders. In addition, the validity of such a study is lower than RCTs.

In the present study, DPP4i were not associated with an increased risk of colon cancer in comparison with other drugs (MH-RR = 0.96, 95% CI = 0.71–1.31, *p* = 0.808). Nevertheless, Femia and colleagues studied the effect of long-term administration of sitagliptin on colon carcinogenesis in rats, at doses comparable to those used in therapeutic settings for humans with diabetes^46^. They found that rats had a significantly lower number of precancerous lesions in the colorectum in those treated with sitagliptin than in controls (*p* = 0.02), which indicated that sitagliptin had a protective effect against colon carcinogensis. The SAVOR-TIMI 53 trial showed a similar protective effect^15^. An *in vitro* study also suggested that DPP4i had anti-cancer property, and sitagliptin was found to be more potent than vildagliptin on inhibiting HT-29 colon cancer cells growth^47^. However, studies reporting that DPP4i had a protective effect on colon cancer were still few. Besides, Wang *et al*.^48^ found that DPP4i antidiabetic treatment did not increase the cancer risk,, and yet saxagliptin and sitagliptin could markedly increase cell migration and invasion of SW480 and HCT116 colon cancer cell lines through activation of nuclear factor E2-related factor 2 (NRF2). Therefore, further investigations also should be necessary to be performed to discuss the role of DPP4i antidiabetic drugs in diabetic patients with cancer.

A few studies also investigated the association between DPP4i and other types of cancer. A retrospective cohort study assessed the risk of prostate cancer associated with sitagliptin, also using the Taiwanese database of the National Health Insurance between 1999 and 2000, and the result of HR = 0.613 (95% CI = 0.493–0.763) showed that sitagliptin could significantly reduce the risk of prostate cancer^49^. Some *in vivo* studies explored the effect of DPP4 on tumorigenesis of the breast, ovary and prostate at the molecular level; however, it was not conclusive whether DPP4 promoted tumorigenesis^50–52^.

The current meta-analysis had several advantages. To the best of our knowledge, the present meta-analysis was the first to evaluate the effect of DPP4i on the risk of cancers based on RCTs. We conducted this meta-analysis using rigorous search and statistical analysis methods to ensure the accuracy and validity of the results. 11 studies were both published in the electronic databases and reported in the trial registry. We checked the data reported in publications against those in the clinical trial registry for consistency. In particular, some published studies we identified from the electronic databases did not report the data of cancer outcome, and we used the NCT codes from the publications to retrieve data on cancer from ClinicalTrials.gov. In this way, we minimized the risk of attrition and reporting bias.

However, several potential limitations in our meta-analysis also should be fully recognized. First, cancer was not the primary endpoint in any of the included trials and was reported as serious adverse events. There were no predefinition or any uniform diagnostic criteria, which could lead to misclassification. Second, the number of trials and the patients included still were insufficient to draw a definitive conclusion on the effect of DPP4i on the risk of cancers. Third, cancer is caused by many factors and cancer development is considerably complicated. In addition, more than a half of available studies the duration of treatment less than 52 weeks, so the duration of trials was still limited, which was inadequate to draw a conclusion on the occurrence of cancer with long-term DPP4i use. More RCTs with longer exposure to such drugs are required. Finally, we performed this study based on summary data; if we conducted the study with patient-level data, the assessment could be more accurate, but it was difficult for us to acquire the relevant data from electronic databases or trial registry.

In conclusion, the results of our meta-analysis showed that there is no significantly increased risk of cancer in patients with type 2 diabetes who are treated with DPP4i than those treated with a placebo or other types of drugs. Given the number of cancer adverse events and the limited duration of trials, there is still a need for large scale RCTs to be conducted to clarify the impact of DPP4i on cancers in the future, at the same time, it is also necessary to conduct pharmacovigilance programs to detect postmarketing AEs of drugs once they approve for marketing, and provide a longitudinal evidence to ensure that patients receive drugs of acceptable benefit-risk profiles.

## Methods

### Search strategy

The electronic databases PubMed, Medline, EMBASE, Web of Science and Cochrane Library were systematically and comprehensively searched to find published articles on randomized clinical trials in humans. The language of literature was limited to English. We combined both MeSH and free text terms to identify all of the relevant articles. Terms used for search were as follows: (1) “sitagliptin,” “vildagliptin,” “saxagliptin,” “alogliptin,” “linagliptin,” “dipeptidyl peptidase 4 inhibitors,” “DPP-4 inhibitors,” or “DPP4i”; (2) “cancer,” “carcinoma,” “tumor,” “neoplasm,” or “adenocarcinomas.” We also identified some completed but still unpublished studies through a search of the www.clinicaltrials.gov website. The literature search was conducted from the inception of each database to 25 July 2016.

### Study selection

Studies were included into this meta-analysis if they met the following criteria: (a) randomized clinical trials; (b) conducted in patients with type 2 diabetes mellitus; (c) compared DPP4i with a placebo or active drugs; (d) with a duration of at least 24 weeks; (e) adverse drug events included cancer. The exclusion criteria were as follows: (a) the participants enrolled in trials had type 1 diabetes or no diabetes; (b) trials had a duration of treatment shorter than 24 weeks, because they could not yield relevant information on the incidence of cancer; (c) adverse drug events did not include tumors, the reported tumors were benign, or trials only reported cancer-specific mortality as an outcome but did not specify the type of cancer; (d) trials with incomplete original data or with two zero events. If several studies with the same population were retrieved, the one with the most complete data was used.

### Data extraction and quality assessment

The title and abstract of studies were screened first, and if they met the inclusion criteria then they were reviewed in detail. The following information was extracted by two of the investigators (Ming Zhao and Xiaolong Lai) from the eligible studies: the first author’s name; year of publication; the national clinical trial (NCT) code; the type of DPP4i and the comparison drug; sample size of the treatment and control groups; the number of events per group; the duration of treatment; the numbers of males and females; mean age; mean glycosylated hemoglobin (HbA1c); mean body mass index (BMI); the duration of type 2 diabetes mellitus; and the type of cancers. Discrepancies between the two investigators were resolved by consensus or adjudication by a third investigator.

The Cochrane Collaboration’s risk of bias tool was used to assess the quality of the involved RCTs. The items for evaluating the bias of studies were classified into the following six domains: (a) random sequence generation (selection bias); (b) allocation concealment (selection bias); (c) blinding of participant, personnel and outcome assessment (performance bias and detection bias); (d) complete outcome data (attrition bias); (e) selective reporting (reporting bias); (f) drug compliance assessment (other bias). For each domain, the risk of bias was divided into low, high and unclear; an answer of “yes” indicated a low risk of bias, and an answer of “no” indicated a high risk of bias, and “unclear” indicated lack of relevant information. Given that cancer adverse event was not the primary outcome in many published RCTs, so there were not reported in the studies but on the website, we combined the published data and the data of cancer events on the www.clinicaltrials.gov website for trials reported in both places to evaluate the quality of involved RCTs.

### Statistical analysis

We followed the preferred reporting items for systematic reviews and meta-analyses (PRISMA) for the reporting of this study^53^. The pooled relative risk (RR) and 95% confident intervals (CIs) calculated by the Mantel-Haenszel method were used to assess the relationship between DPP4i use and the risk of cancers.

Heterogeneity was evaluated using Cochrane *Q* statistic with a *p* < 0.1 considered statistically significant. *I* ^2^ statistic was also used to assess the magnitude of heterogeneity across studies. If *I* ^*2 *^ <50% and *P* > 0.1, there was no significant heterogeneity, and a fixed-effect model was used; otherwise, a random-effect model was applied^54^. Pre-defined subgroup analyses were performed for trials that were stratified by the type of DPP4i (sitagliptin, saxagliptin, alogliptin, or linagliptin), location of cancer (reproductive system, digestive system, integumentary system, urinary system, hematologic system, respiratory system, motor system, endocrine system, nervous system, or cardiovascular system) (First, we combined the same adverse events of cancers and counted the number of studies based on the type of cancer, and then these cancers were classified into 10 types based on the location of cancer and counted the number again, due to a RCT may contain a variety of adverse events of cancers, so there existed duplicate in the final number of studies), drug for comparison (placebo, sulfonylureas, thiazolidinediones, metformin, sodium-glucose co-transporter (SGLT) inhibitors, GLP-1 receptor agonists (GLP-1 RAs), or other antidiabetic agents), trial duration (less than 52 weeks *vs*. 52 weeks or more), mean age (less than 60 years *vs*. 60 years or more), mean HbA1c (less than 8% *vs*. 8% or more), mean BMI (less than 30 kg/m^2^ *vs*. 30 kg/m^2^ or more), or mean diabetes duration (less than 8 years *vs*. 8 years or more).

In order to test the robustness of the results, sensitivity analysis was performed. We carried out the sensitivity analysis by using different statistical models (fixed-effect model vs. random-effect model), using different effect measures (relative risk vs. odds ratio (OR)) and excluding low-quality studies. Finally, publication bias was assessed by funnel plots, and the asymmetry of the funnel plots was evaluated by Begg’s and Egger’s tests; a *p* value ≤ 0.1 was considered statistically significant^55,56^. We also undertook the nonparametric “trim and fill” procedure to further assess the possible effect of publication bias in this meta-analysis. The possibility of hypothetical “missing” studies was considered to exist, and the “trim and fill” method was used to impute their RRs and recalculate a pooled RR that incorporated the hypothetical missing studies as if they actually existed^57^.

Meta-analysis was performed using the Stata software (version 12.0; Stata Corporation, College Station, Texas, USA).

### Availability of materials and data

The datasets generated during and/or analysed during the current study are available from the corresponding author on reasonable request.

## Electronic supplementary material

Supplemental Material
