# Author Correction: Dipeptidyl peptidase-4 inhibitors and cancer risk in patients with type 2 diabetes: a meta-analysis of randomized clinical trials

**DOI:** 10.1038/s41598-017-16510-2

**Published:** 2017-11-24

**Authors:** Ming Zhao, Jiayi Chen, Yanyan Yuan, Zuquan Zou, Xiaolong Lai, Daud M Rahmani, Fuyan Wang, Yang Xi, Qin Huang, Shizhong Bu

**Affiliations:** 1 Runliang Diabetes Laboratory, Diabetes Research Center, School of Medicine, Ningbo University, 315211 Ningbo, China; 2 Department of Public Health, Longsai Hospital, 315200 Ningbo, China; 3 Department of Endocrinology, Changhai Hospital, Second Military Medical University, 200433 Shanghai, China

*Scientific Reports* **7**:8273; doi:10.1038/s41598-017-07921-2; Article published online 15 August 2017

The original version of this Article omitted an affiliation for Ming Zhao. The correct affiliations for Ming Zhao are listed below:

Runliang Diabetes Laboratory, Diabetes Research Center, School of Medicine, Ningbo University, 315211, Ningbo, China.

Department of Public Health, Longsai Hospital, 315200, Ningbo, China.

This has now been corrected in the HTML and PDF versions of this Article, and in the accompanying Supplemental Material.

