# Supplementary material for: Dipeptidyl peptidase-4 inhibitors and cancer risk in patients with type 2 diabetes: a meta-analysis of randomized clinical trials
Source: Sci Rep. 2017 Aug 15;7:8273. doi: 10.1038/s41598-017-07921-2 (PMC5557948; doi:10.1038/s41598-017-07921-2)
Supplement: Supplementary file 1 — Supplemental Material [file 41598_2017_7921_MOESM1_ESM.doc]

# Dipeptidyl peptidase-4 inhibitors and cancer risk in patients with type 2 diabetes: a meta-analysis of randomized clinical trials

Ming Zhao^1,2^, Jiayi Chen^1^, Yanyan Yuan^1^, Zuquan Zou^1^, Xiaolong Lai^1^, Daud M Rahmani^1^, Fuyan Wang^1^, Yang Xi^1^, Qin Huang^3*^, Shizhong Bu^1*^

^1^Runliang Diabetes Laboratory, Diabetes Research Center, School of Medicine, Ningbo University, 315211 Ningbo, China; [ming8260@126.com](mailto:ming8260@126.com) (M.Z.); [13282263303@163.com](mailto:13282263303@163.com) (J.C.); [1511101190@nbu.edu.cn](mailto:1511101190@nbu.edu.cn) (Y.Y.); [zouzuquan@nbu.edu.cn](mailto:zouzuquan@nbu.edu.cn) (Z.Z.); [412676440@qq.com (X.L.)](mailto:412676440@qq.com (X.L.)); [daud.rahmani@yahoo.com (D.M.R.)](mailto:daud.rahmani@yahoo.com (D.M.R.)); [wangfuyan@nbu.edu.cn (F.W.)](mailto:wangfuyan@nbu.edu.cn (F.W.)); [xiyang@nbu.edu.cn (Y.X.)](mailto:xiyang@nbu.edu.cn (Y.X.)); [bushizhong@nbu.edu.cn](mailto:bushizhong@nbu.edu.cn) (S.B.)

^2^Department of Public Health, Longsai Hospital, 315200, Ningbo, China

^3^Department of Endocrinology, Changhai Hospital, Second Military Medical University, 200433 Shanghai, China; [qxinyi1220@163.com](mailto:qxinyi1220@163.com) (Q.H.)

Table S1 Quality assessment of the randomized clinical trials included in the meta-analysis

| **Study** | **NCT code** | **Selection bias** | | **Performance bias** | **Detection bias** | **Attrition bias** | **Reporting bias** | **Other bias** |
| --- | --- | --- | --- | --- | --- | --- | --- | --- |
|  |  | **Random sequence generation** | **Allocation concealment** | **Blinding of participant and personnel** | **Blinding of outcome assessment** | **Complete outcome data** | **Selective reporting** | **Drug compliance assessment** |
| NCT01590771 | NCT01590771 | low | low | low | low | low | low | low |
| NCT01189890 | NCT01189890 | low | low | low | unclear | low | low | low |
| NCT01076088 | NCT01076088 | low | low | low | low | low | low | low |
| NCT00420511 | NCT00420511 | unclear | low | low | unclear | low | low | low |
| NCT01076075 | NCT01076075 | low | low | low | unclear | low | low | low |
| Arjona 2013 | NCT00509262 | low | unclear | low | low | unclear | unclear | low |
| Green 2015 | NCT00790205 | low | low | low | low | low | low | low |
| NCT00885352 | NCT00885352 | unclear | low | low | low | low | low | low |
| NCT00509236 | NCT00509236 | low | low | low | low | low | low | low |
| NCT00095056 | NCT00095056 | low | low | low | unclear | low | low | low |
| NCT00395343 | NCT00395343 | low | unclear | low | unclear | low | low | low |
| NCT00722371 | NCT00722371 | unclear | low | low | unclear | low | low | low |
| NCT00337610 | NCT00337610 | low | unclear | low | low | low | low | low |
| NCT01177384 | NCT01177384 | unclear | unclear | low | unclear | low | low | low |
| NCT00305604 | NCT00305604 | low | low | low | unclear | low | low | low |
| NCT00701090 | NCT00701090 | low | low | low | unclear | low | low | low |
| NCT00449930 | NCT00449930 | low | low | low | low | low | low | low |
| NCT00637273 | NCT00637273 | unclear | unclear | low | low | low | unclear | low |
| NCT01137812 | NCT01137812 | low | low | low | low | low | low | low |
| NCT01098539 | NCT01098539 | unclear | unclear | low | unclear | low | low | low |
| Aschner 2012 | NCT00751114 | low | low | low | low | low | low | low |
| NCT00106704 | NCT00106704 | low | unclear | low | low | low | low | low |
| NCT00881530 | NCT00881530 | unclear | unclear | low | low | low | low | low |
| NCT00482729 | NCT00482729 | unclear | unclear | low | low | low | low | low |
| NCT00086502 | NCT00086502 | unclear | unclear | low | unclear | low | low | low |
| NCT00532935 | NCT00532935 | unclear | unclear | low | low | low | low | low |
| NCT01046110 | NCT01046110 | unclear | unclear | low | low | low | low | low |
| NCT02008682 | NCT02008682 | unclear | unclear | low | unclear | low | low | low |
| Ahren 2014 | NCT00838903 | low | unclear | low | low | unclear | unclear | low |
| NCT00094770 | NCT00094770 | unclear | unclear | low | low | low | low | low |
| Pratley 2012 | NCT00700817 | unclear | unclear | unclear | unclear | unclear | unclear | low |
| NCT00289848 | NCT00289848 | low | low | low | low | low | low | low |
| NCT00813995 | NCT00813995 | low | low | low | low | low | low | low |
| NCT00094757 | NCT00094757 | unclear | low | low | low | low | low | low |
| NCT01519674 | NCT01519674 | unclear | unclear | low | unclear | low | low | low |
| NCT00086515 | NCT00086515 | low | low | low | low | low | low | low |
| NCT01907854 | NCT01907854 | unclear | low | low | low | low | low | low |
| Weinstock 2015 | NCT00734474 | low | low | low | low | unclear | unclear | low |
| NCT00327015 | NCT00327015 | low | low | low | low | low | low | low |
| NCT00121641 | NCT00121641 | unclear | unclear | low | unclear | low | low | low |
| NCT00316082 | NCT00316082 | unclear | unclear | low | unclear | low | low | low |
| NCT00121667 | NCT00121667 | low | low | low | unclear | low | low | low |
| NCT00295633 | NCT00295633 | low | low | low | low | low | low | low |
| NCT00575588 | NCT00575588 | unclear | unclear | low | low | low | low | low |
| NCT00757588 | NCT00757588 | low | low | low | low | low | low | low |
| NCT01128153 | NCT01128153 | unclear | unclear | low | unclear | low | low | low |
| Schernthaner 2016 | NCT01006603 | low | unclear | low | low | unclear | unclear | low |
| NCT00313313 | NCT00313313 | low | low | low | low | low | low | low |
| NCT01006590 | NCT01006590 | low | low | low | low | low | low | low |
| NCT01107886 | NCT01107886 | unclear | unclear | low | low | unclear | low | low |
| NCT00374907 | NCT00374907 | unclear | unclear | low | unclear | low | low | low |
| White 2013 | NCT00968708 | low | low | low | low | low | low | low |
| NCT00286468 | NCT00286468 | low | unclear | low | low | low | low | low |
| NCT00286442 | NCT00286442 | low | unclear | low | low | low | low | low |
| NCT00856284 | NCT00856284 | unclear | unclear | low | unclear | low | low | low |
| NCT00286429 | NCT00286429 | unclear | unclear | low | unclear | low | low | low |
| NCT01263496 | NCT01263496 | unclear | low | low | low | low | low | low |
| NCT00395512 | NCT00395512 | low | low | low | low | low | low | low |
| NCT00707993 | NCT00707993 | unclear | low | low | low | low | low | low |
| NCT00432276 | NCT00432276 | unclear | unclear | low | unclear | low | low | low |
| Mita 2016 | NA | unclear | unclear | low | unclear | low | low | low |
| NCT01084005 | NCT01084005 | low | low | low | low | low | low | low |
| NCT01087502 | NCT01087502 | low | low | low | unclear | low | low | low |
| NCT01734785 | NCT01734785 | unclear | unclear | low | low | low | low | low |
| Bajaj 2014 | NCT00996658 | low | unclear | low | low | low | unclear | low |
| NCT00954447 | NCT00954447 | low | unclear | low | low | low | low | low |
| NCT00798161 | NCT00798161 | unclear | unclear | low | unclear | low | low | low |
| Gallwitz 2012 | NCT00622284 | low | low | low | low | low | low | low |
| NCT01215097 | NCT01215097 | unclear | unclear | low | unclear | low | low | low |
| NCT00621140 | NCT00621140 | low | unclear | low | low | low | low | low |
| NCT00654381 | NCT00654381 | unclear | unclear | low | unclear | low | low | low |
| Barnett 2012 | NCT00740051 | low | unclear | low | unclear | unclear | unclear | low |

NA: not available, NCT: national clinical trial
